# Association between Blood Potassium Level and Recovery of Postoperative Gastrointestinal Motility during Continuous Renal Replacement Therapy in Patient Undergoing Open Abdominal Surgery

**DOI:** 10.1155/2019/6392751

**Published:** 2019-07-02

**Authors:** Yi Yang, Jingjuan Yang, Xiner Yao, Yu Cui, Xiabing Lang, Binbin Wu, Ping Zhang, Jianghua Chen

**Affiliations:** ^1^Kidney Disease Center, The First Affiliated Hospital, College of Medicine, Zhejiang University and Key Laboratory of Kidney Disease Prevention and Control Technology, Zhejiang Province, and The Third Grade Laboratory under the National State, Administration of Traditional Chinese Medicine. 79 Qingchun Road, Hangzhou, China; ^2^Department of Nephrology, The Fourth Affiliated Hospital, College of Medicine, Zhejiang University, N1 Shangcheng Road, Yiwu, China; ^3^Department of Nephrology, The First People's Hospital of Huzhou, 158 Guangchang Road, Huzhou, China

## Abstract

**Background:**

The aim of this study was to identify the blood potassium level beneficial to the postoperative recovery of gastrointestinal motility during continuous renal replacement therapy (CRRT) in patient undergoing open abdominal surgery.

**Materials and Methods:**

538 critically ill patients after open abdominal surgery and receiving CRRT were retrospectively recruited as the study cohort. Demographic and clinical data were recorded along with an evaluation of the postoperative gastrointestinal motility.

**Results:**

Correlation analysis was used to assess the correlation coefficient, and then the variables with correlation coefficient value less than 0.5 were included in the binary logistic regression model. Binary logistic regression model indicated that the postoperative blood potassium level was independently associated with the recovery of gastrointestinal motility (OR=0.109, 95% CI= 0.063 to 0.190, p<0.001). Based on the normal range of blood potassium level, we selected the cut-off point of blood potassium level via Weight of Evidence analysis, which was 4.00 mmol/L. Compared with the patients with insufficient blood potassium levels (plasma potassium concentration < 4.00 mmol/L), those with sufficient blood potassium levels (plasma potassium concentration≥ 4.00 mmol/L) conferred an increase in the rate of 4-day postoperative recovery of gastrointestinal motility (OR= 4.425, 95% CI = 2.933 to 6.667, p<0.001).

**Conclusions:**

Maintaining the blood potassium concentrations at a relatively high level of the normal blood potassium range during CRRT would be beneficial to postoperative recovery of gastrointestinal motility.

## 1. Introduction

Patients undergoing abdominal surgery develop episodes of impaired gastrointestinal motility and even postoperative ileus [1, 2]. Prolonged gastrointestinal paralysis after surgery may result in longer hospital stays and increased medical costs [3]. Electrolyte homeostasis, particularly the blood potassium level, is very important for postoperative recovery of gastrointestinal function [4]. Several studies suggested that hypokalemia was an independent risk factor for postoperative complications, including delayed recovery of gastrointestinal motility, while sufficient potassium supplementation might accelerate recovery of gastrointestinal function [5, 6].

Numerous factors cause potassium disturbances after abdominal surgery, especially in critically ill patients receiving intensive care therapy, including insufficient potassium intake, excessive potassium discharge, hypercatabolism, concomitant acute kidney injury, and therapy related factors [7–9]. Continuous renal replacement therapy (CRRT) is one of the most important methods to maintain electrolyte homeostasis of critically ill patients with or without kidney injury [10, 11]. One of the fundamental goals of the CRRT is to maintain blood potassium levels within a normal range. It is relatively easy to avoid hyperkalemia or hypokalemia, diagnosed according to the classical standard, in the clinical setting when patients are receiving CRRT [12]. However, regarding optimized treatment, maintaining reasonable blood potassium levels to support the postoperative recovery of gastrointestinal motility is the current concern for clinicians. Unfortunately, there are very few reports studying this crucial clinical problem, and the rational goal of blood potassium levels during CRRT remains unclear to date.

In the present study, we retrospectively investigated a patient cohort after abdominal surgery who received CRRT to identify the potential therapeutic goal of the blood potassium level, which is beneficial for the postoperative recovery of gastrointestinal motility.

## 2. Material and Methods

### 2.1. Ethics Approval and Consent to Participate

This study was approved by the Research Ethics Committee of the First Affiliated Hospital, College of Medicine, Zhejiang University, Hangzhou, China, and informed consent was obtained from the patients and/or their guardians.

### 2.2. Patient Selection

Between 1 January 2008 and 30 December 2017, approximately 14,400 patients admitted to the intensive care unit (ICU), emergency ICU, or surgical ICU at the First Affiliated Hospital, College of Medicine, Zhejiang University, were retrospectively reviewed. Among these patients, 538 individuals having undergone open abdominal surgery and receiving CRRT served as the patient cohort for the present study. The patients did not receive premedication. Anaesthesia was induced with propofol 1.5-2 mg/kg and fentanyl 3 *μ*g/kg. Cisatracurium 0.2 mg/kg was given to facilitate orotracheal intubation with a cuffed tube. Anaesthesia was maintained with propofol 6-8 mg/kg and remifentanil 0.5-1 mg/h and oxygen 60%, with positive pressure ventilation in a circle system.

### 2.3. Patient Data

All patient data were extracted from medical records as well as the linked clinical inspection database and blood purification databases of the hospital. We collected the following demographic and clinical information of the patients at admission to ICU and during CRRT treatment: age, sex, operative characteristics, Acute Physiology Chronic Health Evaluation II (APACHE II) and Sequential Organ Failure Assessment (SOFA) scores at the admission to ICU, technical parameters and duration of CRRT, ICU stay time, and daily plasma potassium level. Plasma potassium levels were measured every 4 hours and the mean was adopted to stand for the daily plasma level. Potassium supplementation in the replacement fluid managed to maintain the patients' plasma potassium in the normal range, according to the results of the routine tests. Plasma potassium levels were measured with an ABL800 FLEX analyzer (Radiometer Medical, Copenhagen, Denmark). The Accura Hemofiltration System was used to administer CRRT. Polysulfone or polyamide filters were used for the patients, and the filter was changed when the transmembrane pressure (TMP) of the filter was greater than 250 mmHg. Central venous access was obtained using catheters of 11.5-Fr or 13.5-Fr × 16 cm, 11.5-Fr or 13.5-Fr ×19.5 cm. Replacement fluid was delivered into the extracorporeal circuit at a predilution/postdilution ratio of 2:1. Anticoagulation was performed according to each patient's condition with unfractionated heparin, low-molecular-weight heparin, heparin-free, or citrate.

### 2.4. Variables

Observation indices of gastrointestinal motility included bowel sounds, flatus, or defecation. The first gastrointestinal motility recovery time was defined as any of the following episodes: (1) the time of first bowel sound, defined as the time from the completion of the surgery to the first bowel sound; (2) the first flatus time, defined as the time from the completion of surgery to the first spontaneous flatus after surgery; and (3) the first defecation time, defined as the time from the completion of surgery to the first spontaneous defecation after the surgery. In the present study cohort, the first gastrointestinal motility recovery time showed an abnormal distribution, and the median time was 4 days. Therefore, we used gastrointestinal motility recovery during 4-day period and lack of recovery at 4 days after the completion of surgery as the primary endpoints. The plasma potassium concentrations before the primary endpoints were recorded to calculate the means, adopted as the postoperative blood potassium levels.

### 2.5. Statistical Analysis

Statistical analysis was performed using R Language software (R i386 3.5.1, New Zealand). A* p*-value less than 0.05 was considered statistically significant. A univariate comparison was performed to compare variables between two groups using unpaired t-tests for continuous variables and a *χ*2 tests or Fisher's exact tests for categorical variables. Correlation analysis was used to determine the correlation coefficient between variables. A correlation coefficient value greater than 0.5 was considered to be relevant. Binary logistic regression analysis was used to identify the independent contribution of prognostic factors to the prediction of gastrointestinal motility recovery 4 days after the completion of surgery. When constructing the multivariate model, the variables with correlation coefficient value less than 0.5 were included in the binary logistic regression model. Odds ratios with 95% confidence intervals (CIs) were used to estimate the association between the independent variables and the dependent variable. Kaplan–Meier analysis was used to evaluate the association between blood potassium level and postoperative gastrointestinal motility.

## 3. Results

**Figure 1:**
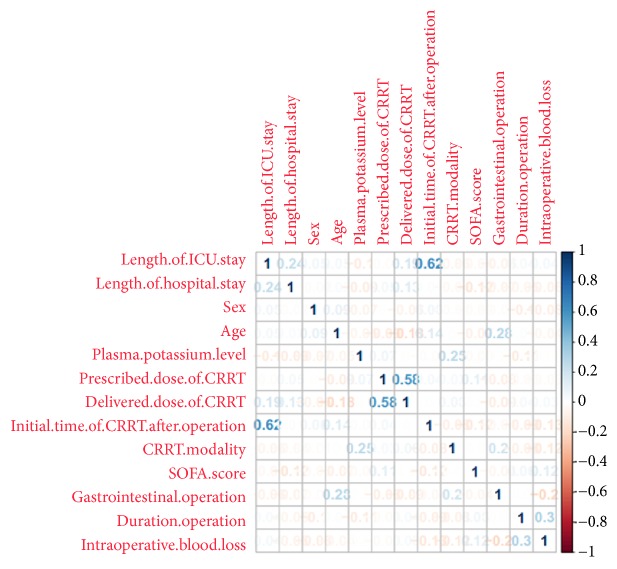
*Correlation analysis between variables*. It demonstrated that correlation coefficient between length of ICU stay and initial time of CRRT after operation was 0.62 and between delivered dose of CRRT and prescribed dose of CRRT was 0.58. A correlation coefficient value greater than 0.5 was considered to be relevant.

The median first gastrointestinal motility recovery time was 4 days. According to the primary endpoint described in Material and Methods, all recruited patients were divided into the following groups: the recovery group was defined as the time of gastrointestinal motility recovery ≤ 4 days after the completion of surgery, and gastrointestinal motility recovery > 4 days was defined as the nonrecovery group. The baseline demographics and clinical characteristics of the patients are summarized in Table 1. The mean age (±SD) for patients of the study cohort was 53.50 ± 15.95 years, and 364 patients (67.66%) were male. A total of 156 patients (29.00%) underwent gastrointestinal operations. Correlation analysis between variables demonstrated that correlation coefficient between length of ICU stay and initial time of CRRT after operation was 0.62 and between delivered dose of CRRT and prescribed dose of CRRT was 0.58 (Figure 1). Therefore, the variables with correlation coefficient value less than 0.5 were included in the binary logistic regression model. These factors indicated that the postoperative blood potassium level was independently associated with the recovery of gastrointestinal motility (OR=0.109, 95% CI= 0.063 to 0.190,* p*<0.001, Table 2).

**Figure 2:**
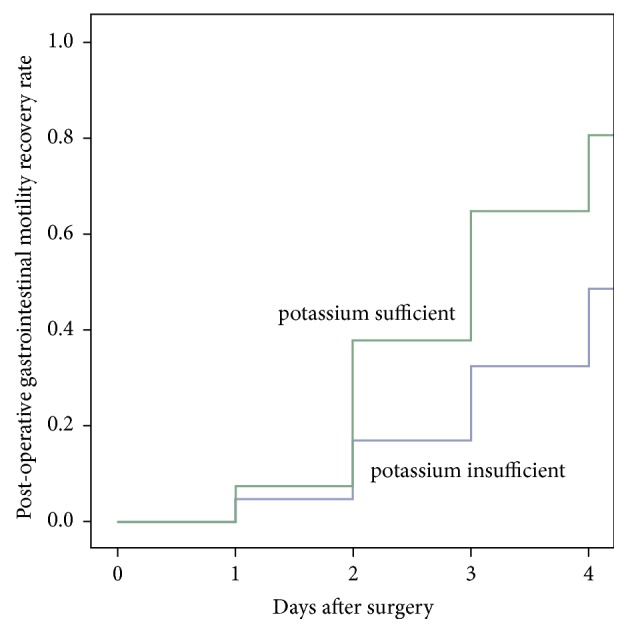
*Kaplan–Meier analysis between blood potassium level and postoperative gastrointestinal motility*. It indicated that, compared with the patients with insufficient blood potassium levels (plasma potassium concentration < 4.00 mmol/L), those with sufficient levels (plasma potassium concentration ≥4.00 mmol/L) had higher rates of 4-day postoperative recovery of gastrointestinal motility (p<0.001).

**Figure 3:**
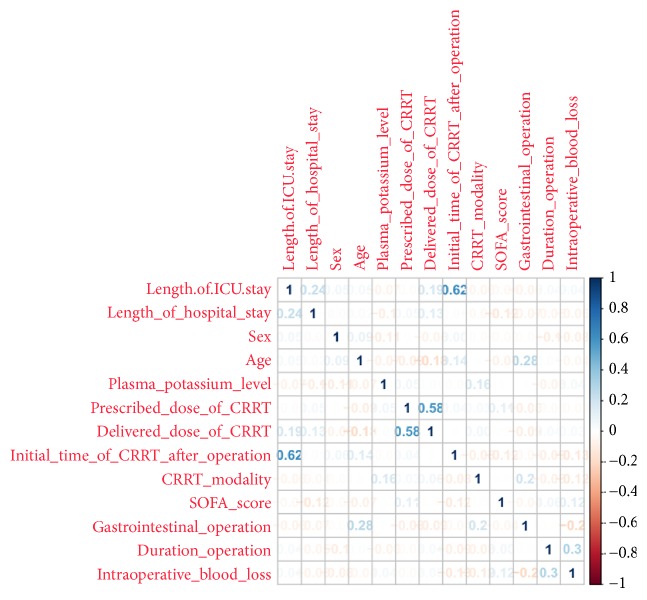
*Correlation analysis between variables*. Among them, blood potassium concentration was defined as insufficient group and sufficient group. It demonstrated that correlation coefficient between length of ICU stay and initial time of CRRT after operation was 0.62 and between delivered dose of CRRT and prescribed dose of CRRT was 0.58. A correlation coefficient value greater than 0.5 was considered to be relevant.

Based on the normal range of blood potassium level, we selected the cut-off point of blood potassium level* via* Weight of Evidence analysis, which was 4.00 mmol/L. Therefore, we divided the total recruited patients into following two groups: potassium insufficient group was defined as mean postoperative plasma potassium concentration < 4.00 mmol/L before the primary endpoint and postoperative plasma potassium concentration ≥ 4.00 mmol/L was defined as the sufficient group. Kaplan–Meier analysis indicated that postoperative gastrointestinal motility would recover more quickly in the potassium sufficient patients (p<0.001, Figure 2). After correlation analysis between variables, among which blood potassium concentration was defined as insufficient group and sufficient group, initial time of CRRT after operation, delivered dose of CRRT, potassium group, and other nonrelevant variables were included in the binary logistic regression model (Figure 3). Binary logistic regression model demonstrated that, compared with the patients with insufficient blood potassium levels (plasma potassium concentration < 4.00 mmol/L), those with sufficient blood potassium levels (plasma potassium concentration≥ 4.00 mmol/L) conferred an increase in the rate of 4-day postoperative recovery of gastrointestinal motility (OR= 4.425, 95% CI = 2.933 to 6.667, p<0.001, Table 3).

The baseline demographics and clinical characteristics of the potassium insufficient and sufficient groups are summarized in Table 4. As to the length of hospital stay, that of potassium insufficient patients was significantly longer than that in potassium sufficient patients. Some technical parameters of CRRT were compared between two groups in addition to the rate of postoperative gastrointestinal motility recovery. Compared with the potassium insufficient patients, the potassium sufficient individuals more frequently received CRRT, including the hemodialysis method. There was no significant difference in terms of dose of CRRT between the sufficient and insufficient groups.

## 4. Discussion

Electrolyte homeostasis, particularly the blood potassium level, is crucial for normal gastrointestinal function. Maintenance of blood potassium concentrations at a sufficient level is the cornerstone of stable transmembrane potential that permits normal muscle function, including gastrointestinal motility [13]. In the general population, the regular range for serum potassium levels is typically between 3.5 and 5.3 mmol/L, whereas the optimal range of potassium concentration in patients differs. For example, several studies indicated that a blood potassium level relatively higher than the normal blood potassium range was associated with low incidence of all-cause mortality in hemodialysis patients [14–16]. Adequate potassium supplementation might accelerate the recovery of gastrointestinal motility after abdominal surgery [6]. The data of the present study also demonstrated that a blood potassium level higher than the normal range was beneficial for postoperative recovery of gastrointestinal motility, and maintaining the plasma potassium concentration ≥ 4.0 mmol/L was associated with a significant increase in the rate of 4-day postoperative recovery of gastrointestinal motility.

In the postoperative setting, multiple factors might meditate disturbances of the blood potassium levels, causing delayed postoperative recovery of gastrointestinal motility. It is a challenge to maintain the electrolyte concentrations at reasonable levels. CRRT is one of the widely used therapeutic strategies to manage fluids, electrolytes, and acid-base balance in critically ill patients. To date, the indications for the initiation of CRRT are far beyond typical clinical conditions, including fluid overload, hyperkalemia, and severe acidosis [17]. To maintain internal environmental homeostasis is the fundamental therapeutic goal of medical treatment, and CRRT may be adopted in many cases even without acute kidney injury [10]. In fact, a portion of the total patients in the present study received CRRT according to the extended application indication. Based on the technical features of CRRT, hyperkalemia is relatively easy to manage in clinical practice. No patients developed hyperkalemia episodes during CRRT in the present study. Alternatively, insufficient blood potassium levels, even hypokalemia, are more frequent in critically ill patients, especially caused by CRRT itself. It was reported in the ATN study that the incidence of hypokalemia was 4.5% in the low-intensity group and 7.5% in the high-intensity group [18]. In the RENAL trial, the incidences of hypokalemia in the low- and high-intensity groups were 24.4% and 23.4%, respectively [19]. Since the updates of technologies of CRRT, in particular, one of the most important methods that we currently have in clinical management is the ability to modify potassium concentration of dialysate or replacement fluid [12]. Theoretically, clinicians can prescribe regimens for any therapeutic blood potassium level goal. Therefore, information regarding a reasonable goal, as least in part, becomes a fundamental element of the entire strategy for CRRT management. Unfortunately, data regarding the reasonable blood potassium level in critically ill patients during CRRT are very rare. Our data provided evidence for a potential therapeutic goal of blood potassium concentration in the scenario described above.

Evaluation of gastrointestinal motility is important but difficult in clinical practice. Several parameters are usually used to evaluate gastrointestinal motility, including bowel sounds, flatus, defecation, and ability to tolerate solid food [20]. In clinical trials, the time to the return of bowel sounds, first flatus, and defecation are often used as primary and/or secondary outcome measures [21]. In the present study, the recovery of gastrointestinal motility was defined as any of the following three situations: return of bowel sounds, spontaneous flatus, or spontaneous defecation. It is important to underscore that these parameters are difficult to assess accurately in practice. Theoretically, passing stool or flatus may reflect rectal emptying rather than the recovery of effective gastrointestinal motility [20]. Furthermore, the complicated situation of critically ill patients may restrict the collection or recording of the information, for example, mechanical ventilation, sedation, disturbances of consciousness, and parenteral nutrition. So the parameters for the evaluation of gastrointestinal motility adopted in the present study might introduce bias. Therefore, one should be cautious in drawing conclusions based on the noted plasma potassium concentration of 4.00 mmol/L (presented as the cut-off point of Weight of Evidence analysis). Nevertheless, our data might in part fulfill the objective of the present study by exploring the trend that keeping the blood potassium concentration at a relatively high level of the normal blood potassium range during CRRT is beneficial to the postoperative recovery of gastrointestinal motility.

Electrolyte management via CRRT in clinical practice depends on the selection of technical parameters, especially the modality and therapeutic dose [8, 9, 17]. Our data also indicated that the modality adopted with the hemodialysis method more frequently maintained the blood potassium levels at sufficient concentrations. Nevertheless, we did not find a therapeutic dose effect on the maintenance of blood potassium levels. The present data implied that adding a hemodialysis method to the CRRT modality might be beneficial to maintain the patient's blood potassium levels at a particular therapeutic goal level due to the innate technical character of the hemodialysis method [22], in particular, in the clinical setting using mass fluid replacement.

The current study had several limitations. The retrospective study design decreased the power of the conclusions. We defined gastrointestinal motility recovery during 4-day period and nonrecovery at 4 days after the completion of surgery as the primary endpoints, according to the median of the first gastrointestinal motility recovery time of the study cohort. One should be cautious to use the median as the surrogate for the regular postoperative gastrointestinal motility recovery time. As described above, the definition we adopted for the evaluation of gastrointestinal motility might cause bias. In addition, various clinical settings may affect postoperative recovery of gastrointestinal function in critically ill patients; therefore, we could never exclude the potential that factors other than blood potassium levels during CRRT could impact the study outcome. A rational prospectively randomized controlled trial should be designed to resolve this bias.

## 5. Conclusions

We retrospectively investigated a patient cohort after open abdominal surgery receiving CRRT and found that blood potassium level during CRRT was significantly associated with postoperative recovery of gastrointestinal motility. Maintaining blood potassium concentrations at relatively high levels of the normal blood potassium range (plasma potassium concentration ≥ 4.00 mmol/L) was beneficial to the postoperative recovery of gastrointestinal function. Compared with the patients with insufficient blood potassium level (plasma potassium concentration <4.00 mmol/L), those with sufficient levels (plasma potassium concentration ≥4.00 mmol/L) had an increase in the rate of 4-day postoperative recovery of gastrointestinal motility (OR= 4.425, 95% CI = 2.933 to 6.667,* p*<0.001). Our data suggested that maintaining blood potassium concentrations at relatively high levels of the normal blood potassium range during CRRT would be beneficial to postoperative recovery of gastrointestinal motility, suggesting a potential therapeutic blood potassium level goal for the recovery of postoperative gastrointestinal motility during CRRT in patient undergoing open abdominal surgery.

## Figures and Tables

**Table 1:** Baseline demographic and clinical characteristics of the patients^a^.

Characteristics	Patients (n=538)	Recovery (n=336)	Nonrecovery (n=202)
*Male sex (*%)	364 (67.66)	224 (66.67)	140 (69.31)
*Mean age (y)*	53.50±15.95	53.61±16.17	53.31±15.61
*Operative characteristics*			
Gastrointestinal operation (%)	156 (29.00)	101 (30.06)	55 (27.23)
No-gastrointestinal operation (%)	382 (71.00)	235 (69.94)	147 (72.77)
Duration of operation(mean, min)	229.68±140.79	219.63±139.38	246.41±141.87
Intraoperative blood loss (median, ml)	400.00 (100.00-1025.00)	300.00 (100.00-1000.00)	500.00 (100.00-1200.00)
*Length of ICU stay (median, day)*	7.00 (4.00-13.00)	6.00 (4.00-11.00)	8.00 (5.00-15.00)
*Length of hospital stay (median, day) *	27.00 (13.00-40.00)	24.00 (11.00-38.00)	32.00 (17.00-45.00)
*APACHE II score (mean)*	16.80±7.78	16.83±7.39	16.77±8.48
*SOFA score (mean)*	8.64±3.99	8.43±3.92	8.99±4.10
*Initial time of CRRT after operation (median, day)*	1.00 (0.00-3.00)	1.00 (0.00-3.00)	1.00 (0.00-3.00)
*CRRT modality*			
CVVH (%)	464 (86.25)	283 (84.23)	181 (89.60)
CVVHD/CVVHDF (%)	74 (13.75)	53 (15.77)	21 (10.40)
*Prescribed dose of CRRT (mean in first 72 hours, ml/kg/h)*	39.25±10.62	38.86±10.64	39.92±10.56
*Delivered dose of CRRT (mean in first 72 hours, ml/kg/h)*	29.65±13.30	29.62±14.04	29.70±11.98
*Plasma potassium level (mmol/L)*	4.00±0.47	4.14±0.48	3.78±0.34
*Survival rates (*%)	289 (53.71)	181 (53.86)	108 (53.47)

a Recovery is defined as the time of gastrointestinal motility recovery ≤ 4.0 days after the completion of surgery, and > 4 days considered as non-recovery.

CVVH stands for continuous veno-venous hemofiltration, CVVHD stands for continuous veno-venous hemodialysis, and CVVHDF stands for continuous veno-venous hemodiafiltration.

Plasma potassium level was defined as the mean plasma potassium concentration before the primary endpoints.

*p*-value represents recovery cohort vs. nonrecovery cohort.

**Table 2:** Variables included in the binary logistic regression analysis and hazard ratios.

Variable	OR (95% CI)	*P*
Male sex	1.267 (0.834-1.923)	0.267
Mean age (y)	1.000 (0.987-1.013)	0.990
Gastrointestinal operation	1.139 (0.718-1.808)	0.581
Duration of operation (mean, min)	1.001 (0.999-1.002)	0.232
Intraoperative blood loss (median, ml)	1.000 (1.000-1.000)	0.803
Length of hospital stay (median, day)	1.003 (0.998-1.009)	0.258
SOFA score (mean)	1.038 (0.989-1.091)	0.134
Initial time of CRRT after operation (median, day)	0.970 (0.935-1.007)	0.108
CRRT modality	0.901 (0.476-1.704)	0.748
Delivered dose of CRRT (mean in first 72 hours, ml/kg/h)	0.997 (0.983-1.012)	0.716
Plasma potassium level (mmol/L)	0.109 (0.063-0.190)	<0.001

**Table 3:** Variables included in the binary logistic regression analysis and hazard ratios^b^.

Variable	OR (95% CI)	*P*
Male sex	1.295 (0.860-1.951)	0.216
Mean age (y)	0.999 (0.986-1.011)	0.821
Gastrointestinal operation	1.099 (0.699-1.726)	0.684
Duration of operation (mean, min)	1.001 (1.000-1.002)	0.134
Intraoperative blood loss (median, ml)	1.000 (1.000-1.000)	0.803
Length of hospital stay (median, day)	1.003 (0.998-1.008)	0.220
SOFA score (mean)	1.040 (0.992-1.091)	0.107
Initial time of CRRT after operation (median, day)	0.975 (0.941-1.010)	0.152
CRRT modality	1.145 (0.630-2.079)	0.657
Delivered dose of CRRT (mean in first 72 hours, ml/kg/h)	0.998 (0.984-1.012)	0.773
With sufficient plasma potassium level	4.425 (2.933-6.667)	<0.001

b potassium sufficient group was defined as the patient's mean postoperative plasma potassium level ≥ 4.00 mmol/L before the primary endpoints defined in the present study.

**Table 4:** Baseline demographic and clinical characteristics of the potassium insufficient or sufficient group^c^.

Characteristics	Patients (n=538)	potassium insufficient (n=305)	potassium sufficient (n=233)	*p*
*Male sex (*%)	364 (67.66)	193 (63.28)	171 (73.39)	0.013
*Mean age (y)*	53.50±15.95	54.47±16.06	52.22±15.74	0.104
*Operative characteristics*				
Gastrointestinal operation (%)	156 (29.00)	88 (28.85)	68 (29.18)	0.933
Nongastrointestinal operation (%)	382 (71.00)	217 (71.15)	165 (70.82)	0.933
Duration of operation (mean, min)	229.68±140.79	237.19±137.89	219.85±144.20	0.157
Intraoperative blood loss (mean, ml)	400 (100-1025)	500 (100-1000)	300 (100-1150)	0.365
*Length of ICU stay (median, day)*	7.00 (4.00-13.00)	8.00 (5.00-14.00)	6.00 (3.00-12.00)	0.114
*Length of hospital stay (median, day) *	27.00 (13.00-40.00)	30.00 (17.00-45.00)	23.00 (10.00-35.00)	0.026
*APACHE II score (mean)*	16.80±7.78	16.67±8.23	16.97±7.23	0.689
*SOFA score (mean)*	8.64±4.00	8.63±4.00	8.64±3.99	0.967
*Initial time of CRRT after operation (median, day)*	1.00 (0.00-3.00)	1.00 (0.00-3.00)	1.00 (0.00-2.00)	0.668
*CRRT modality*				<0.001
CVVH (%)	464 (86.25)	278 (91.15)	186 (79.83)	
CVVHD/CVVHDF (%)	74 (13.75)	27 (8.85)	47 (20.17)	
*Prescribed dose of CRRT (mean in first 72 hours, ml/kg/h)*	39.25±10.62	38.79±10.76	39.86±10.42	0.242
*Delivered dose of CRRT (mean in first 72 hours, ml/kg/h)*	29.65±13.30	29.49±14.31	29.87±11.87	0.743
*Postoperative gastrointestinal motility recovery in 4.0 days (*%)	336 (62.45)	148 (48.52)	188 (80.69)	<0.001
*Survival rates (*%)	289 (53.71)	159 (52.13)	130 (55.79)	0.399

c potassium insufficient group was defined as the patient's mean postoperative plasma potassium level < 4.00 mmol/L before the primary endpoint defined in the present study, and postoperative plasma potassium level ≥ 4.00 mmol/L was considered as the sufficient group.

CVVH stands for continuous veno-venous hemofiltration, CVVHD stands for continuous veno-venous hemodialysis, and CVVHDF stands for continuous veno-venous hemodiafiltration*. p*-value represents potassium insufficient cohort vs. potassium sufficient.

## Data Availability

The data used to support the findings of this study are available from the corresponding author upon request.

## References

[1] Collins T. C., Daley J., Henderson W. H., Khuri S. F. (1999). Risk factors for prolonged length of stay after major elective surgery. *Annals of Surgery*.

[2] Geltzeiler C. B., Rotramel A., Wilson C., Deng L., Whiteford M. H., Frankhouse J. (2014). Prospective study of colorectal enhanced recovery after surgery in a community hospital. *JAMA Surgery*.

[3] Johnson M. D., Walsh R. M. (2009). Current therapies to shorten postoperative ileus. *Cleveland Clinic Journal of Medicine*.

[4] Lobo D. N., Bostock K. A., Neal K. R., Perkins A. C., Rowlands B. J., Allison S. P. (2002). Effect of salt and water balance on recovery of gastrointestinal function after elective colonic resection: a randomised controlled trial. *The Lancet*.

[5] González-Fajardo J., Mengibar L., Brizuela J., Castrodeza J., Vaquero-Puerta C. (2009). Effect of postoperative restrictive fluid therapy in the recovery of patients with abdominal vascular surgery. *European Journal of Vascular and Endovascular Surgery*.

[6] Lu G., Xu L., Zhong Y., Shi P., Shen X. (2014). Significance of serum potassium level monitoring during the course of post-operative rehabilitation in patients with hypokalemia. *World Journal of Surgery*.

[7] Basheeth N., O'Cathain E., O'Leary G., Sheahan P. (2014). Hypocalcemia after total laryngectomy. *The Laryngoscope*.

[8] McMahon G. M., Mendu M. L., Gibbons F. K., Christopher K. B. (2012). Association between hyperkalemia at critical care initiation and mortality. *Intensive Care Medicine*.

[9] Joannidis M., Druml W., Forni L. G. (2010). Prevention of acute kidney injury and protection of renal function in the intensive care unit. Expert opinion of the Working Group for Nephrology, ESICM. *Intensive Care Medicine*.

[10] Ronco C., Ricci Z., De Backer D. (2015). Renal replacement therapy in acute kidney injury: controversy and consensus. *Critical Care*.

[11] Wang Y., Haines T. P., Ritchie P. (2014). Early mobilization on continuous renal replacement therapy is safe and may improve filter life. *Critical Care*.

[12] Karaboyas A., Zee J., Brunelli S. M. (2017). Dialysate potassium, serum potassium, mortality, and arrhythmia events in hemodialysis: results from the dialysis outcomes and practice patterns study (DOPPS). *American Journal of Kidney Diseases*.

[13] Ardalan M., Golzari S. E. J. (2015). An integrated view of potassium homeostasis. *The New England Journal of Medicine*.

[14] Pun P. H., Lehrich R. W., Honeycutt E. F., Herzog C. A., Middleton J. P. (2011). Modifiable risk factors associated with sudden cardiac arrest within hemodialysis clinics. *Kidney International*.

[15] Kovesdy C. P., Regidor D. L., Mehrotra R. (2007). Serum and dialysate potassium concentrations and survival in hemodialysis patients. *Clinical Journal of the American Society of Nephrology*.

[16] Pun P. H., Middleton J. P. (2017). Dialysate potassium, dialysate magnesium, and hemodialysis risk. *Journal of the American Society of Nephrology*.

[17] Joannidis M., Forni L. G. (2011). Clinical review: timing of renal replacement therapy. *Critical Care*.

[18] Palevsky P. M., Zhang J. H., O'Connor T. Z. (2008). Intensity of renal support in critically ill patients with acute kidney injury. *The New England Journal of Medicine*.

[19] Fayad A. I., Buamscha D. G., Ciapponi A. (2016). Intensity of continuous renal replacement therapy for acute kidney injury. *Cochrane Database of Systematic Reviews*.

[20] Van Bree S. H. W., Bemelman W. A., Hollmann M. W. (2014). Identification of clinical outcome measures for recovery of gastrointestinal motility in postoperative ileus. *Annals of Surgery*.

[21] Toyomasu Y., Mochiki E., Morita H. (2011). Mosapride citrate improves postoperative ileus of patients with colectomy. *Journal of Gastrointestinal Surgery*.

[22] Schneider A. G., Bellomo R., Bagshaw S. M. (2013). Choice of renal replacement therapy modality and dialysis dependence after acute kidney injury: a systematic review and meta-analysis. *Intensive Care Medicine*.

